# Pulsatility Index and Hypoxia Inducible Factor-1α Expression Predict the Clinical Response after External Radiation in Patients with Stage IIB to IVA Cervical Cancer

**DOI:** 10.31557/APJCP.2019.20.7.2073

**Published:** 2019

**Authors:** I Gde Sastra Winata, Yudi Mulyana Hidayat, Gatot Nyarumenteng Winarno, Dodi Suardi, Setiawan Soetopo, Ketut Suwiyoga

**Affiliations:** 1 *Division of Gynecologic Oncology, Department of Obstetrics and Gynecology, *; 2 *Deparment of Radiotherapy, dr. Hasan Sadikin Hospital/Faculty of Medicine, Padjajaran University, Bandung, *; 3 *Division of Gynecologic Oncology, Department of Obstetrics and Gynecology, Sanglah Hospital/Faculty of Medicine, Udayana University, Denpasar, Bali, Indonesia.*

**Keywords:** pulsatility index- resistance index- hypoxia-inducible factor-1α- clinical response- external radiotherapy

## Abstract

**Objective:**

To evaluate the ability of pulsatility index (PI), resistance index (RI), and hypoxia inducible factor-1α (HIF-1α) expression in predicting the clinical response after radiation in patients with cervical cancer.

**Methods:**

A prospective cohort was carried on in Department of Obstetric and Gynecology Dr. Hasan Sadikin Hospital/ Faculty of Medicine, Padjadjaran University, during the period of July 2017 to March 2018 which include 51 samples with stage IIB to IVA cervical cancer. Tumor perfusion and oxygenation were evaluated using color Doppler ultrasound indices (pulsatility index and resistance index) and the expression of hypoxia inducible factor-1α (HIF-1α). The clinical response was assessed 2 months after external radiation.

**Result:**

Among 51 patients, 31 patients demonstrated good response and 20 patients demonstrated poor response to radiation. The mean value of PI was significantly lower in patients who demonstrated good response as compared to patients with poor response (0.84±0.916 vs. 1.70±1.260, p = 0.004). The mean value of RI did not differ significantly (0.29±0.112 vs. to 0.36±0.189 p =0.173). HIF-1α expression was significantly lower in patients who demonstrated good response as compared to patients with poor response (1.83±1.529 vs. 6.55±2.625, p = 0.0001). In multivariate model, PI and HIF-1α expression both predicted the clinical response after radiation.

**Conclusion:**

PI and HIF-1α expression predict the clinical response after radiation in patients with cervical cancer.

## Introduction

Cervical cancer ranks the fourth most common cancer in women, after breast, colorectal, and lung cancer. Its worldwide incidence is estimated to be 17 in 100.000 women (Organization, 2006; Hacker, 2015). In Indonesia, cervical cancer is the second most common cancer among women and its annual incidence keeps increasing (Parkin et al., 2005). Survival rate of cervical cancer largely depends on the stage at diagnosis. In Indonesia, around 40 to 44% cases are diagnosed at stage IIIB (Koh et al., 2019). Radiation is the mainstay of treatment for advance disease that localized within the pelvis, i.e stage IIB to IVA. Disease free survival after radiation is comparable to surgery performed in early stage (Koh et al., 2019). Furthermore, radiation can decrease the local recurrence after surgery in high risk patients. Response to radiation depends on histologic type, grade of differentiation, size, vascularization, and location of the tumor (Perez et al., 1998; Eifel, 2015). More differentiated, bigger size and less vascularize tumor are more resistant to radiation. Tissue oxygenation in cervical cancer also predict response to radiation. Sensitivity to radiation in highly oxygenated tissue is three times higher than the hypoxic or anoxic tissue (Perez et al., 1998).

Several methods to determine the degree of tissue oxygenation are available, such as: oxygen electrode, functional imaging, and histopathology examination (Sarti, 2011). The use of oxygen electrode is the gold standard since it can measure tissue oxygenation directly. However, this method is invasive, expensive, and not readily available. Functional imaging to visualize the microcirculation of cervical cancer is another method to determine the degree of tissue oxygenation and neovascularization. Color Doppler ultrasonography is one of functional imaging that is widely used. It offers several advantages since it is a non-invasive method, easier and simpler, less expensive, and more readily available (Alcazar et al., 2003). Cervical perfusion is best evaluated through the blood flow from the cervical branch of uterine artery. However, it poses technical difficulty in identifying the cervical branch of uterine artery. Neovascularization of cervical cancer is best evaluated through intratumoral approach using color Doppler ultrasonography. The most commonly used Doppler indices are resistance index (RI) and pulsatility index (PI) (Dodampahala et al., 2016).

Intratumoral blood flow correlated to several tumor characteristics, such as grade, histology type, volume, and stage. Tumor with poor differentiation, higher volume, and more advance stage present with higher vascularization characterized by decreased RI and PI (Huang et al., 2013; Mangla and Singla, 2015). RI decreases significantly after radiation which indicate decreased perfusion and oxygenation to cervical tissue after radiation (Alcazar et al., 2003; Mangla and Singla, 2015). Recent study suggested that RI and PI were increased significantly after radiation in patients with cervical cancer stage IIA to IIIB who demonstrated complete and partial response as compared to patients who do not demonstrated any response (Mangla and Singla, 2015). PI less than 1.475 has 89.5% sensitivity and 93.5% specificity in detecting increased vascularization in cervical cancer (Dodampahala et al., 2016).

Molecular biomarker also plays an important part as the predictor of tissue oxygenation, especially in cervical cancer. Recent studies focused on the role of Hypoxia-Inducible Factor 1α (HIF-1α) in cervical cancer (Kim et al., 2013; Huang et al., 2014; Xu et al., 2016). HIF-1α is a transcription factor that plays important part in the regulation of gene expression under hypoxic condition such as in embryogenesis, cardiovascular disease, and carcinogenesis (Sowter et al., 2003). It also take part in regulation of gene expression that involved in energy metabolism, survival, proliferation, apoptosis, and tumor invasiveness (Schmid et al., 2004). Loss of HIF-1α is associated with decreased tumor growth, vascularization, and energy metabolism. Meanwhile, increased HIF-1α decreases tumor latency, increases vascular density, tumor volume, permeability, and growth (Hockel et al., 1999). HIF-1α is also associated with lymph node metastasis and stage of cervical cancer (Huang et al., 2014). The spatiotemporal dinamic of adaptive response to radiation induced by HIF-1α expression is demonstrated by Schwartz et al (Schwartz et al., 2011). HIF-1α expression is highly associated with stromal ischemia. In ischemic condition, there is a minimal effect of radiation in inhibiting transcriptional activity of HIF-1α. Furthermore, there are increased expression of HIF-1α and VEGF production in respond to vascular dysfunction induced by radiation. Increased HIF-1α expression is associated with reduced 5-year survival rate. Increased HIF-1α is an independent predictor of poor disease-free survival and overall survival (Seeber et al., 2011).

HIF-1α is a potential biomarker that can predict response to radiation. HIF-1α expression before radiation is significantly higher in patient with partial response as compared to patient with complete response. Higher pretreatment HIF-1α expression is also found in patient with pelvic lymph node involvement as compared to patient without pelvic lymph node involvement. HIF-1α expression also predict the pelvic lymph node involvement with 66.67% sensitivity and 88.89% specificity (Seeber et al., 2011). Therefore, HIF-1α is highly associated with progressivity, response to radiation, as well as the prognosis of cervical cancer. This study is aimed to evaluate the ability of pulsatility index (PI), resistance index (RI), and hypoxia inducible factor-1α (HIF-1α) expression in predicting the clinical response after radiation in patients with cervical cancer.

## Materials and Methods

*Design*

A prospective cohort study was carried on in Obstetric and Gynecology Department, and Pathology Anatomy Department of Hasan Sadikin Hospital/Faculty of Medicine Padjajaran University, Bandung, Indonesia, during July 2017 to March 2018.

*Participants and recruitment*

Patients with cervical cancer stage IIB to IVA who were set to receive external radiation and had given their written consent to participate in this study were recruited into the study population. Patients who were ineligible to receive transrectal ultrasound (e.g. due to hemorrhoid, perianal pain, or anal stenosis) were excluded from the study population. Patient who were lost during follow up and whose tissue sample did not meet the standard criteria for histopathology examination were also excluded. Using formula for sample size calculation in cohort study (α 0.05, β 0.80, Zα 1.645, Zβ 0.842, Confidence Interval 95%), a sample of 44 patients was targeted for the study. A total of 51 patients participated in this study. Although we aimed to include patients with stage IIB to IVA, we were only able to acquired patients with stage IIB to IIIB.

*Variables and measurement Cervical tissue oxygenation*

Cervical tissue oxygenation were evaluated using two approach, i.e. evaluation of intratumoral cervical vascularity using Doppler indices (RI and PI) and tumoral HIF-1α expression using imunohistochemistry staining of cervical tissue sample obtained from biopsy. RI and PI were measured using transrectal approach (GE Logic P3 probe E8CS transrectal, Pulse Wave (PW) Doppler mode). Transrectal approach was preferred since transvaginal approach was associated with the risk of active bleeding during manipulation of the cervix as bleeding from the cervix will interfere the Doppler evaluation.

Patients was placed in lithotomy position. The location, size, and the extend of the tumor invasion to the pelvic wall, parametrium and the surrounding organs were assessed clinically. Tumor size was presented in its largest diameter (cm). A transrectal probe was then inserted to evaluate cervical vascularization. The probe was tilted no more than 60 degree to the axis of the blood vessel. After Pulse Wave (PW) Doppler was activated, peripheral and central RI and PI and their mean values were collected. PI was calculated from the difference between peak systolic and end diastolic velocity divided by the mean velocity.

**Figure 1:**
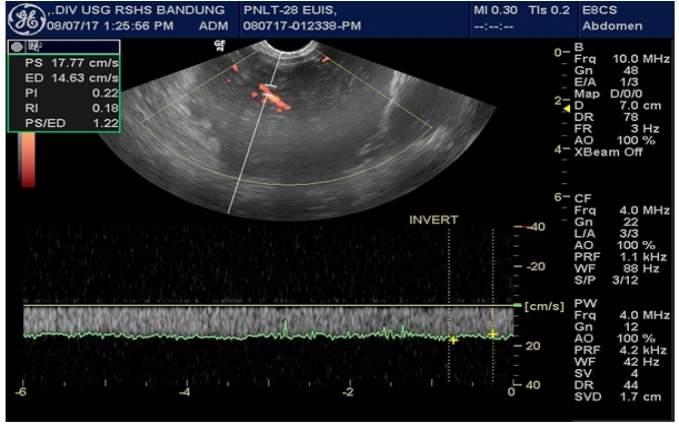
Intratumoral Pulsatility Index (PI) and Resistance Index (RI) Evaluation

RI was calculated from the difference between peak systolic and end diastolic velocity divided by the diastolic velocity (Figure 1).

**Figure 2:**
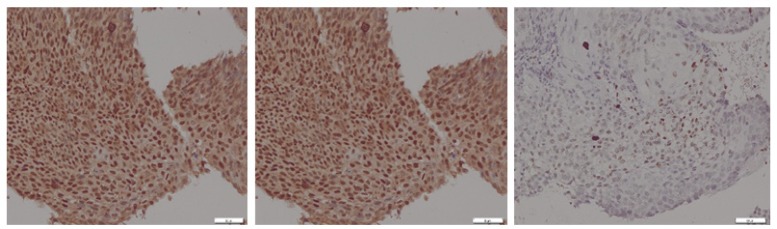
Immunostaining for Hypoxia-inducible Factor-α (HIF-α) Expression Showing Strong (left), Moderate(center), and Weak Staining (right)

The degree of HIF-1α expression was assessed based on the intensity and distribution of the staining. The staining intensity was categorized as 0 (negative), 1+ (weak), 2+ (moderate), and 3+ (strong), while staining distribution was categorized in percentage, i.e. 0 (≤ 5%), 1+ (6-25%), 2+ (26-50%), 3+ (51-75%) and 4+ (> 75%)

(Figure 2). A score was then calculated for each sample by multiplying the intensity score and the distribution score (range 0-12) (Masoud and Li, 2015).

*Clinical response after external radiation*

Patients received 25 cycle of external radiation (200 cGy) followed with 10 cycles (200 cGy) of smaller area as replacement for brachytherapy. The protocols was adopted due to the limitations to perform brachytherapy. Two month after radiation, clinical response was assessed. Clinical response was determined using WHO criteria (complete, partial, stable, and progressive disease) (Tirkes et al., 2013). Complete response was classified as good response, while partial response, stable, and progressive disease were classified as poor response. Tumor size was determined clinically by measuring the largest diameter of the tumor.

*Statistical analysis*

Data were analyzed using SPSS version 24.0. Univariate analysis was used to generate frequencies and percentages of categorical variables. Continuous variables were presented as mean ± SD. Numeric data were tested for normality using Saphiro-Wilk test. Independent t-test was used to compare continuous variables. Chi-square and Kolmogorov Smirnov were used to compare categorical variables. For analysis, we compare RI, PI, HIF-1α expression according to the clinical response after external radiation. The association was determined using Spearman correlation test. Multivariate analysis using binary logistic regression was used to determine the predictor of clinical response to external radiation. Level of statistical significance (p-value) was set at 0.05.

*Ethics approval*

Ethics approval from Research Ethics Committee, Faculty of Medicine Padjadjaran University/dr. Hasan Sadikin Hospital, Bandung, Indonesia, was obtained before commencement of the study.

## Results

*Background characteristics*

Table 1 summarizes the background characteristics of the study population. We did not found significant differences in tumor grading, histologic type, and tumor size among the good and poor responder. Therefore, the evaluation of PI and RI did not affected by the tumor characteristics.

*PI, RI, and HIF-1α expression*

Table 2 and 3 summarizes the difference of PI, RI, and HIF-1α expression according to the clinical response after radiation. Mean PI and HIF-1α score were significantly lower in patients with good response as compared to patients with poor response (0.84±0.916 vs. 1.70±1.260, p = 0.004; 1.83±1.529 vs. 6.55±2.625, p = 0.0001). Mean RI was not differ significantly between the good and the poor responder. Interestingly, we found a negative and moderate correlation between PI and HIF-1α among patients with poor response (R -0.521, p = 0.018).

**Table 1:** Background Characteristics of the Study Population

Variable	Clinical response		p value
	Good	Poor	
	N=31	N=20	
Age (years)			0.655
Mean±SD	50.67±10.051	51.90±8.534	
Median	48.00	51.50	
Range (min-max)	31.00-75.00	40.00-73.00	
Parity			0.972
< 3	11 (35.5%)	7 (35.0%)	
> 3	20 (64.5%)	13 (65.0%	)
Stage			0.891
IIB	13 (41.9%)	8 (40.0%)	
IIIB	18 (58.1%)	12 (60.0%	)
Histology type			0.593
Squamous Cell Ca	27 (87.1%)	13 (65.0%	)
Adenocarcinoma	4 (12.9%)	3 (15.0%)	
Others	0 (0.0%)	4 (20.0%)	
Differentiation			1.000
Well-differentiated	4 (12.9%)	4 (20.0%)	
Moderately differentiated	19 (61.3%)	12 (60.0%	)
Poorly differentiated	8 (25.8%)	4 (20.0%)	
Tumor size (cm)			0.243
Mean±SD	5.27±1.493	5.75±1.251	
Median	5.00	5.50	
Range (min-max)	2.50-8.50	4.00-9.00	

SD, Standard deviation. p-value only compares mean and proportion (%)

**Table 2:** Difference in Pulsatility Index (PI) and Resistance Index (RI) According to Clinical Response after External Radiation

Doppler index	Clinical response		p value
	Good	Poor	
	N=31	N=20	
Pulsatility index (PI)			
Mean±SD	0.84±0.916	1.70±1.260	0.004
Median	0.47	1.57	
Range (min-max)	0.08-4.32	0.13-5.38	
Resistance index (RI)			
Mean±SD	0.29±0.112	0.36±0.189	0.173
Median	0.33	0.34	
Range (min-max)	0.06-0.55	0.10-0.71	

SD, Standard deviation. p-value only compares mean.

**Table 3:** Difference in HIF-1α Expression According to Clinical Response after External Radiation

HIF-1α	Clinical response		p value
	Good	Poor	
	N=31	N=20	
Score			
Mean±SD	1.83±1.529	6.55±2.625	0.0001
Median	2.00	6.00	
Range (min-max)	0.00-6.00	4.00-12.00	

p-value only compares mean.

*The correlation between PI, RI, HIF-1α expression and the clinical response after radiation*

Table 4 summarizes the correlation between PI, RI, HIF-1α expression and the clinical response after radiation. Strong and positive correlation were observed for PI and HIF-1α expression (R=0.411, p=0.003; R=0.801, p=0.0001, respectively). RI did not correlate with the clinical response after radiation.

*Multivariate analysis*

Table 5 summarize the multivariate analysis of study variables. PI and HIF-1α expression both predict clinical response after radiation. We calculate a formula to predict the clinical response after radiation based on the result of logistic regression analysis. The formula was y = -10.293+ 1.937 PI + 1.897 HIF-1α. The clinical response was predicted to be good if the y value was negative while poor response was predicted if the y value was positive.

**Table 4:** Correlation between PI, RI, HIF-1α Expression and the Clinical Response after External Radiation

Variable	R	p value
Pulsatility Index (PI)	0.411	0.003
Resistance Index (RI)	0.131	0.359
HIF-1α expression	0.801	0.0001

R, Correlation coeeficient

**Table 5:** Logistic Regression Analysis PI, HIF-1α Expression and the Clinical Response after External Radiation

Variable	Odd Ratio	Confidence Interval (95%) Lower-Upper
PI	6.937	1.986-22.356
HIF-1α	6.663	1.238-38.875
Constant		

PI, > 0.71 vs. < 0.71; HIF-1α expression, >5.00 vs. < 5.00. Reference group, PI < 0.07, HIF-1α expression < 5.00

## Discussion

In this study, we demonstrate the association between PI, RI, HIF-1α and the clinical response after external radiation in patients with stage IIB to IVA cervical cancer. Mean value of PI and HIF-1α were significantly lower in patients who demonstrated good response after radiation. We also found that RI did not differ significantly between patient with good and poor response after radiation. Our study suggests that PI and HIF-1α expression can predict the clinical response after radiation in patients with cervical cancer. HIF-1α expression was a stronger predictor as compared to PI. We were able to constructed a logistic regression model that incorporate the value of PI and HIF-1α expression as follows: Ln P/1-P= -10.293+ 1.937 PI + 1.897 HIF-1α.

The degree of tissue oxygenation have been proposed as one of the predictors of clinical response to radiation. Highly oxygenated tumor is three times more sensitive to radiation than the hypoxic or anoxic tumor (Xu et al., 2016). Tissue oxygenation and perfusion depend on 2 main factors, i.e. blood flow to the tissue and the peripheral resistance. PI can be used to evaluate the cervical blood flow and should be measured within the central and peripheral zone of the tumor (Alcazar et al., 2003; Alcazar et al., 2004). It can predict blood flow variability and neovascularization of the cervical cancer. Lower intratumoral PI is associated with higher vascularization and tissue oxygenation distal from the sampling site. Thus, tumor with lower PI should have better response to radiation (Huang et al., 2013). We demonstrate that patients with lower PI demonstrated better response to radiation. Our results are in accordance with results from several studies that investigate the role of PI in predicting clinical response to radiation. Dodampahala et al investigated PI in stage IIB to IVA cervical cancer through cervical branch of uterine artery and the intratumoral blood vessels (Dodampahala et al., 2016). They found that PI less than 1.475 gave 89.5% sensitivity and 93.5% specificity in predicting increased vascularization. Moreover, PI increases significantly after radiation (Alcazar et al., 2003; Mangla and Singla, 2015).

Increased intratumoral blood flow is also associated with decreased RI (Mangla and Singla, 2015). RI less than 0.5 gives 81% sensitivity and 93% specificity in predicting neovascularization in cervical cancer (Sarti, 2011). Kerimoglu et al reported that RI increased significantly after radiation in stage IIB to IVA cervical cancer (Kerimoglu et al., 2006). Mangla et al reported that RI increased significantly in patients with complete and partial response after radiation as compared to patients without response (Mangla and Singla, 2015). However, we did not detect any significant difference of RI value between patients with good and poor response after radiation. The new blood vessels in malignant tumor are characterized by discontinued endothelial layer and basal membrane and absence of tunica media that result in decreased peripheral resistance. They are also characterized by the dysregulation of mechanism that control the luminal diameter. These characteristics result in relatively constant vascular resistance, and hence, constant RI. The presence of arterio-venous shunt within the tumor also contributes to low impedance blood flow (Alcazar et al., 2003). These mechanisms may explain why the RI value did not differ significantly between patients with good and poor response in our study.

We found that HIF-1α expression was significantly lower in patients with good response to radiation as compared to patients with poor response. Xu et al reported that HIF-1α expression was higher in patients with partial response as compared to patients with complete response (Xu et al., 2016). Low HIF-1α expression is associated with better response to radiation (Seeber et al., 2011). Kim et al reported that higher HIF-1α expression was lower 5-year survival rate (Kim et al., 2013). Increased HIF-1α was an independent predictor of lower disease-free survival and overall survival rate (Seeber et al., 2011). Thus, HIF-1α is a potential biomarker to predict clinical response to radiation. Tissue hypoxia is one of the responsible mechanisms underlying the failure of radiation therapy in cervical cancer (Xu et al., 2016). Tissue hypoxia is highly associated increased HIF-1α expression. In hypoxic condition, the effect of radiation in inhibiting transcriptional activity of HIF-1α is minimal. Hypoxia triggers increased expression of HIF-1α and production of VEGF (Xu et al., 2016). Higher HIF-1α expression will in turn increases production of proangiogenic factor. On the other hand, tumor with low HIF-1α and proangiogenic factors expression are more sensitive to microvascular damage after radiation (Kung et al., 2000).

Our study has several limitations. Our sample size was small, thus the power to detect significant differences between the poor and good responder may be limited. Furthermore, our follow up was relatively short. The ability of PI, RI, and HIF-1α expression to predict long term clinical response after radiation such as survival rate cannot be concluded. Further research is needed to compare different approach to evaluate cervical tumor oxygenation, for example spectral approach, as well as to increase the validity of the prediction model.

In conclusion, the degree of tissue oxygenation predicts clinical response after radiation in cervical cancer. We suggested the use of Doppler ultrasonography and HIF-1α expression to help predicts the clinical response in patients who are candidates for receiving radiation.

*Funding statement*

The authors declares no funding resources.
